# Supplementary material for: MetaRibo-Seq measures translation in microbiomes
Source: Nat Commun. 2020 Jun 29;11:3268. doi: 10.1038/s41467-020-17081-z (PMC7324362; doi:10.1038/s41467-020-17081-z)
Supplement: Supplementary file 10 — Supplementary Data 7 [file 41467_2020_17081_MOESM10_ESM.zip › File2/Confidence_High_Taxonomy/305665_out.krona.html]

Javascript must be enabled to view this page.

members
magnitude
magnitudeUnassigned
count
unassigned
taxon
rank

305665\_out

18

18
superkingdom
2

976
18
phylum

200643
18
class

order
18
171549

18
family
171552

838
18
genus


SRS011134\_contig\_number\_8697SRS013687\_contig\_number\_28862SRS016541\_contig\_number\_5943SRS017307\_contig\_number\_4295SRS017849\_contig\_number\_contig-100\_308.308SRS019808\_contig\_number\_18498SRS022609\_contig\_number\_14140SRS023914\_contig\_number\_13030SRS049959\_contig\_number\_17309SRS050752\_contig\_number\_contig-100\_55.132526SRS053356\_contig\_number\_38032SRS054059\_contig\_number\_contig-100\_7671.43024SRS064757\_contig\_number\_21489SRS1041116\_contig\_number\_8263SRS104912\_contig\_number\_13631SRS144537\_contig\_number\_19645SRS147614\_contig\_number\_1385
165179
17
species

1262929

SRS049896\_contig\_number\_9689
1
species
